# The risk factors involved in airway mucus plug in children with ADV Pneumonia

**DOI:** 10.1186/s12890-023-02756-2

**Published:** 2023-11-17

**Authors:** Jing-jing Huang, Lin Yuan, Zhi-qiang Zhuo, Ming-zhen Li, Xing-dong Wu

**Affiliations:** Department of Infection, Xiamen Children’s Hospital (Children’s Hospital of Fudan University at Xiamen), NO.92 Yibin Road, Huli District, Xiamen, 361006 Fujian China

**Keywords:** Adenovirus pneumonia, Children, Fibreoptic bronchoscopy, Mucus plug, Risk factors

## Abstract

**Background:**

The risk factors for mucus plug in children with adenovirus (ADV) pneumonia.

**Methods and materials:**

A retrospective analysis was conducted of children diagnosed ADV pneumonia and underwent fiberoptic bronchoscopy admitted to the Xiamen Children's Hospital from September 2018 to September 2021.The patients were divided into a mucus plug group (39 cases) and a non-mucus plug group (53 cases). The children's data including sex, age, clinical presentation, laboratory test parameters, imaging and bronchoscopic data were collected. The risk factors for the development of airway mucus plug were analysed by multifactorial logistic regression.

**Results:**

There were no statistically significant differences in sex, age, fever, hospitalization days, mixed infection, white blood cells (WBC) count, percentage of neutrophils (NE%), C-reactive protein(CRP), and D-dimer (all *P* > 0.05); Thermal range, procalcitonin (PCT), lactate dehydrogenase (LDH), Pleural effusion and associated decreased breath sounds was significantly higher in mucus plug group than in non-mucus plug group, and the differences were statistically significant (all *P* < 0.05); multifactorial logistic regression analysis showed that the duration of fever, PCT, and LDH were independent risk factors for the formation of mucus plugs. The critical values of ROC curves were pyroprocedure ≥ 6.5 d, PCT ≥ 0.705 ng/ml and LDH ≥ 478.5 U/L.

**Conclusion:**

Duration of fever, PCT and LDH levels were the independent risk factors for the formation of an airway mucus plug in children with ADV pneumonia.

## Introduction

Adenovirus (ADV) is an enveloped double-stranded DNA virus with seven subtypes and about 100 genotypes, and is an important pathogen for immunocompromised people. ADV infections are more common in children than in adults because of their immune function is not yet complete, and they are more common in children aged 6 months to 5 years old [1, 2]. ADV is highly contagious, and some infected children may have no clinical symptoms or present with only mild symptoms of upper respiratory tract infection, however,others may still develop pulmonary infections, with a severe clinical manifestation, many extrapulmonary complications, and a tendency for severe cases to be left with chronic airway and lung diseases [3]. From the end of 2018 to 2019, due to the epidemic of ADV infection in southern China, the proportion of patients treated with fiberoptic bronchoscopy and alveolar lavage increased accordingly because of the increase in patients with severe ADV pneumonia leading to pulmonary atelectasis.And it was found that in the acute stage of children with ADV pneumonia, different degrees of damage to the mucosa of the airway can occur, such as mucosal congestion and swelling, mucus secretion increases, and some of the formation of mucus plugs or even plastic bronchial formation to block the airway, and the emergence of inflammatory If the obstruction of the airway is not relieved in time, it can cause poor tracheo-bronchial ventilation, and increase the chance of occlusive bronchiolitis, bronchiectasis and hyaline lung in the long term, which affects the lung function and quality of life of the children [4–6]. Currently, there is no specific treatment, high morbidity and mortality rate, and poor prognosis, so it needs to attract great clinical attention [7, 8]. In this study, we analyzed the risk factors of ADV pneumonia complicated with mucous plug, aiming at the early detection of mucous plug, timely relevant treatment, stopping the progression of the disease and reducing the occurrence of sequelae.

## Data collection and methodology

### General data

Children hospitalized in Xiamen Children's Hospital from September 2018 to September 2021 who met the diagnosis of ADV pneumonia and underwent fiberoptic bronchoscopy were selected for the study. Inclusion criteria was as follows: (1) Age from 28d to 14 years old, gender is not limited; (2)Children fulfilling the diagnostic criteria of ADV pneumonia as described in the diagnostic and treatment specifications for ADV pneumonia in children (2019 version) jointly issued by the National Health Commission of the People's Republic of China and the State Administration of Traditional Chinese Medicine [3]; (3) Diagnosis of ADV infections: positive test of seven respiratory viral antigens on nasopharyngeal swab, sputum, or alveolar lavage of the child; some cases were diagnosed by second-generation sequencing diagnosis;(4) Lung imaging showed lobar or segmental atelectasis, with inflammation involving 1/3 of the lung lobes or more, and there were indications for fiberoptic bronchoscopy and bronchoalveolar lavage. (5) Previously of good health with no other underlying disease or recurrent respiratory infections. Informed consent was obtained from the parents or other guardians of the children. Exclusion criteria was as follows:(1) Those with clearly mixed other viral, bacterial, fungal and other pathogenic infections were excluded. (2) Those with incomplete clinical data unable to complete statistical analysis were excluded.

**Fig. 1:**
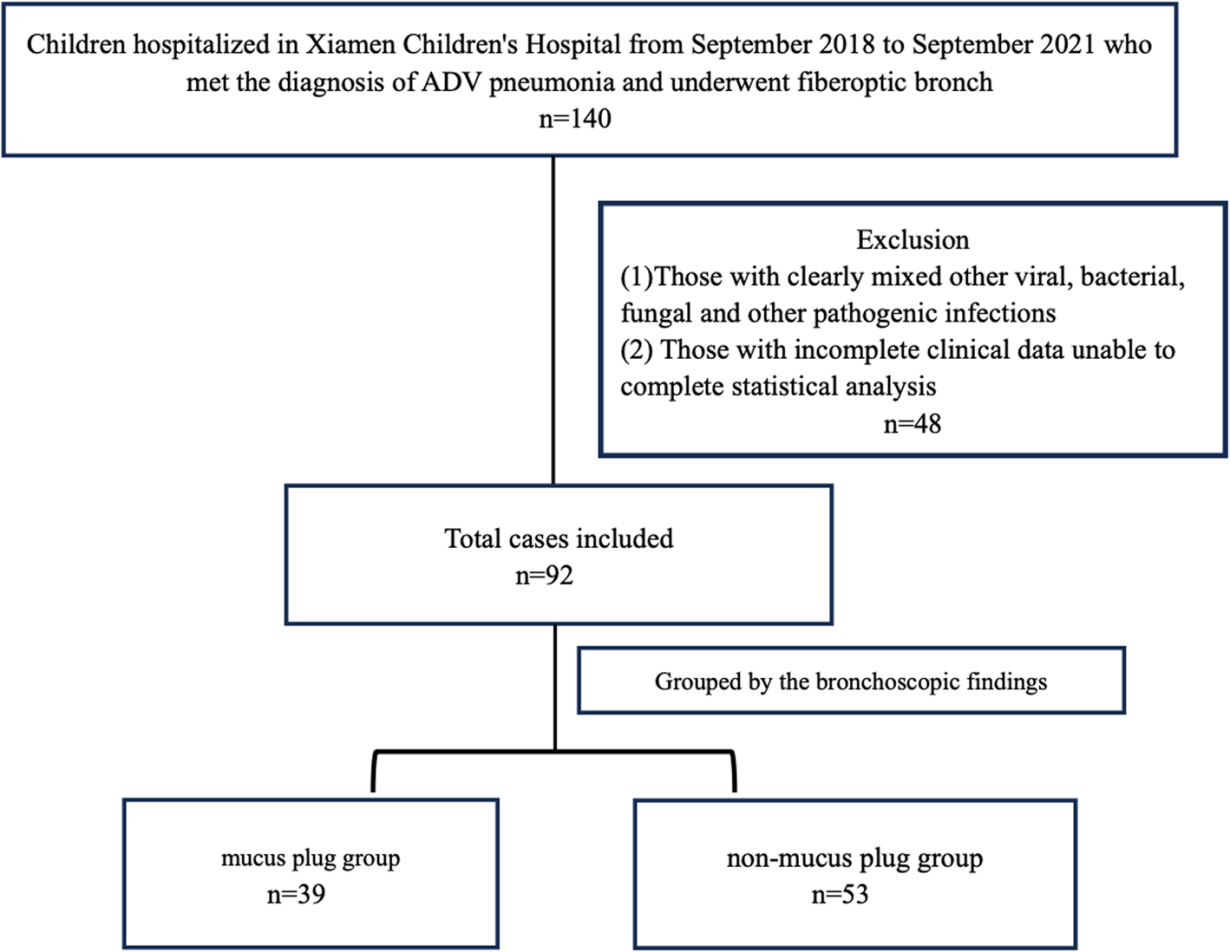
Flowchart of the study population

Finally, a total of 92 patients were included according to the inclusion and exclusion criteria. There were 58 males and 34 females, aged 9 months-9 years and 4 months. The patients were divided into two groups based on the bronchoscopic findings. There were 39 cases in the group with an airway mucus plug and 53 cases in the non-mucus plug group, The flow chart of the research subjects is shown in Fig. 1. In the group with mucus plugs, the bronchial lumens of one or more lung segments were found to be obstructed by viscous secretions, which could not be easily aspirated. The group without mucous plugs showed only mucosal hyperaemia, oedema and a small amount of thin and flocculent secretions in the lumen. These secretions did not form a mucus plug and were easily removed by lavage or aspiration.

### Methods

#### Aetiological tests

Two sputum specimens were collected from the children by aseptic negative pressure aspiration within 24 h of admission, and one of which was sent for detection of seven respiratory viral antigens (including influenza virus A/B, parainfluenza virus 1/2/3, respiratory syncytial virus, and adenovirus) by direct immunofluorescence, the other was sent for sputum culture. Qualified sputum samples had squamous epithelial cells < 10 cells/low-power field and leukocytes > 25 cells/low-power field. Unqualified samples were re-collected.

Ninety-two children were admitted to the hospital and their venous blood was collected for testing for serological antibodies against Mycoplasma pneumoniae and venous blood from two other sites were collected for bacterial culture and susceptibility testing.

#### Fiberoptic bronchoscopy and alveolar lavage

The patients were instructed to fast for 6 h and avoid drinking water for 4 h before the procedure. Fibreoptic bronchoscopy with different external diameters was selected according to the age of the patient. The respiratory tract mucosa was anesthetized by entering the respiratory tract through the nose; the nasal cavity, voice valve, trachea, bronchial findings and bronchial mucosa, were observed step by step. Alveolar lavage fluid from the lesion was collected for bacterial culture and seven respiratory aetiologies were monitored. This was followed by lavage of the diseased lung segment with 0.9% saline and the mucus thrombi that were difficult to remove by local lavage were removed with foreign body forceps or cell brushes. Finally, the fibreoptic bronchoscope was slowly extracted.

#### Analysis of the clinical data of the two groups

(1) General data such as age, sex, presence of fever with cough, duration of fever, peak temperature, length of hospital stay, and presence of decreased breath sounds were recorded. (2) Laboratory examinations and imaging results like white blood cells (WBC), percentage of neutrophils (NE%), C-reactive protein (CRP), lactate dehydrogenase (LDH), D-dimer, blood culture, sputum culture, serological test for Mycoplasma pneumoniae antibodies, aetiological tests for the seven respiratory viruses, alveolar lavage fluid culture and pulmonary imaging were documented. These results indicated the site of pulmonary solid opacities and the presence of combined pleural effusions.(3) Fiberoptic bronchoscopic presentation with or without mucus plugs or plastic bronchial formation. (4) Status of the treatment and the outcomes (including recurrences) were recorded.

#### Statistical processing

SPSS version 25.0 statistical software was used for data processing. Discrete variables were expressed as rates or component ratios, and the *x*^2^ test was used for comparison between the groups (the variables included: sex, whether the peak fever temperature was greater than 40℃, whether there was a decrease in respiratory sounds, and whether there was combined pleural effusion). Continuous variables, conforming to the normal distribution (i.e. CRP and NE%), were expressed as mean ± standard deviation and were subjected to independent samples t-test; all the other variables that were not normally distributed were expressed as median and quartiles (P25: 25^th^ percentile; P75: 75^th^ percentile), and the nonparametric rank sum test was used for comparison between the two groups. *P* < 0.05 was considered statistically significant. The clinically and statistically significant univariates were subjected to the multivariate logistic regression analysis to derive the independent predictors. Receiver operating characteristic (ROC) curves were used to derive cut-off values for each independent predictor.

## Results

### Clinical characteristics

Among the 92 children included in the study, there were 39 patients with mucus plugs. Out of these, 20 were male and 19 were female, with the age at onset ranging from 4 months and 15 days to 9 years and 4 months. There were 53 patients without mucus plugs. Out of these, 38 were males and 15 were females, with the age at onset ranging from 7 months and 3 days to 9 years. There were no significant differences between the two groups in terms of sex or age at onset (Table 1).

**Table 1:** Comparison of the clinical characteristics of the children with ADV pneumonia in the group with mucus plugs and the group without mucus plugs

Grouping	N	Age (months) [M(P_25_,P_75_)]	Gender (male/female)	Fever course (d) [M(P_25_,P_75_)]	Peak temperature ≥ 40℃ [N (%)]	Days of hospital stay (d) [M (_P25_, _P75_)]	Diminished breath sounds [N (%)]
Group without mucus plugs	53	34(25,57.5)	38/15	6(5,8)	37(69)	12(10,15.5)	18(33)
Group with mucus plugs	39	33(19,58)	20/19	8(7,10)	34(87)	13(9,20)	26(66)
t/Z/*x*^2^ values		-0.830	4.019	-4.618	3.847	-0.919	9.63
P-value		0.407	0.052	< 0.01	0.05	0.358	0.002

*N* number, *M* median, *P25* 25th percentile, *P75* 75th percentile, *t* student t-test, *Z* z-test, *x*^2^ chi-square

The children in both the groups had fever and cough. In the mucus plug group, the proportion of children with fever duration and decreased breath sounds at the lesion site was higher than that of the control group, and the differences were statistically significant (all *P* < 0.05).The differences between the two groups in terms of fever peaks and days of hospitalization were not statistically significant, shown in Table 1.

### Laboratory examinations and imaging findings

The levels of PCT and LDH for the group with mucus plugs were significantly higher than those for the group without (*P* < 0.05) The differences in the WBC, NE% and the levels of CRP, and D-dimer were not statistically significant between the two groups, as shown in Table 2. Segmental or multisegmental subsegmental solid opacities, predominantly in the two lower lungs, were seen on lung imaging in both groups of children.In the group with mucus plugs, there were 16 cases of lesions in the left lower lobe, 2 cases involving the left upper lobe, 3 cases involving the right upper lobe, and 13 cases involving the right lower lobe, and 5 cases of multiple solid lesion failure in both lungs. In the group without the mucus plugs, there were 18 cases of lesions in the left lower lobe, 10 cases involving the right upper lobe and 13 cases involving the right lower lobe, and 9 cases of multiple solid lesion failure in both lungs. The proportion of children with a concurrent pleural effusion was significantly higher in the group with mucus plugs than in the group without (*P* < 0.05) (Table 2).

**Table 2:** Comparison of the laboratory investigations and imaging results of children with ADV pneumonia in the group with mucus plugs and the group without mucus plugs

Grouping	N	WBC [× 10^9^/L M(P_25_,P_75_]	NE [%, x ± s]	CRP[mg/L, x ± s]	PCT[ng/ml, M(P_25_,P_75_]	LDH[U/L, M(P_25_,P_75_]	D-dimer mg/l, M(P_25_,P_75_)	Pleural effusion [N (%)]
Group without mucus plugs	53	7.4 (5.79,10.37)	13.08 ± 16.3	58.2 ± 16.25	0.22 (0.07,0.69)	420 (324,618)	0.72 (0.457,1.02)	20(37)
Group with mucus plugs	39	5.82 (4.17,9.23)	16.6 ± 19.4	61.22 ± 14	1.25 (0.71,4.09)	701 (535,1219)	0.86 (0.404,1.77)	25(64)
t/Z/*x*^2^ values		-1.505	-0.768	-0.928	-4.377	-4.531	-0.755	6.251
*P*-value		0.132	0.109	0.56	< 0.01	< 0.01	0.451	0.012

*N* number, *WBC* white blood cells, *NE%* proportion of neutrophils, *CRP* C-reactive protein, *PCT* procalcitonin, *LDH* lactate dehydrogenase, *M* median, *P*_*25*_ 25^th^ percentile, *P*_*75*_ 75^th^ percentile, *t* student t-test, *Z* z-test, *x*^2^ chi-square

### Findings of the fiberoptic bronchoscopy

**Fig. 2:**
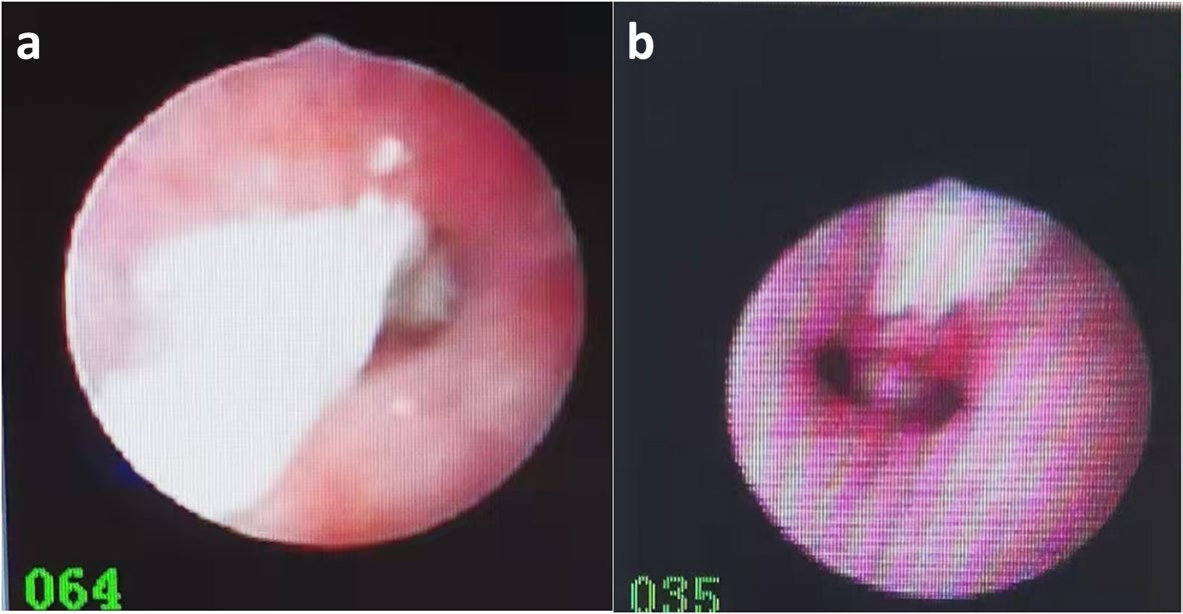
**a** and **b** Trachea blocked by mucus plugs

**Fig. 3:**
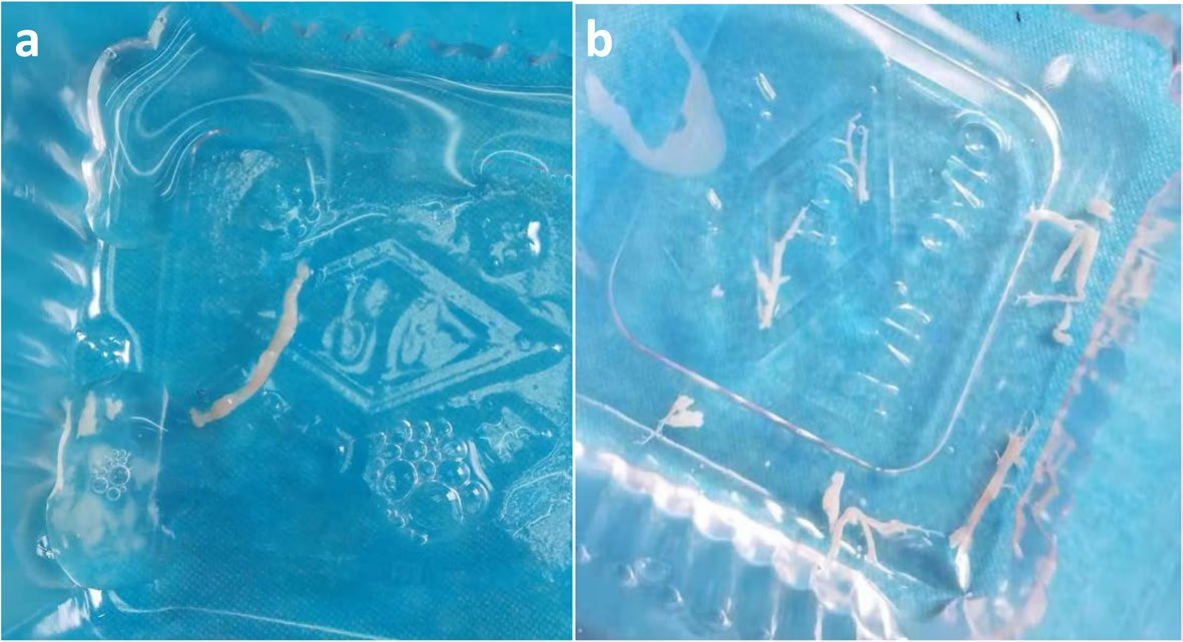
**a** and **b** Display of various removed plastic casts

**Fig. 4:**
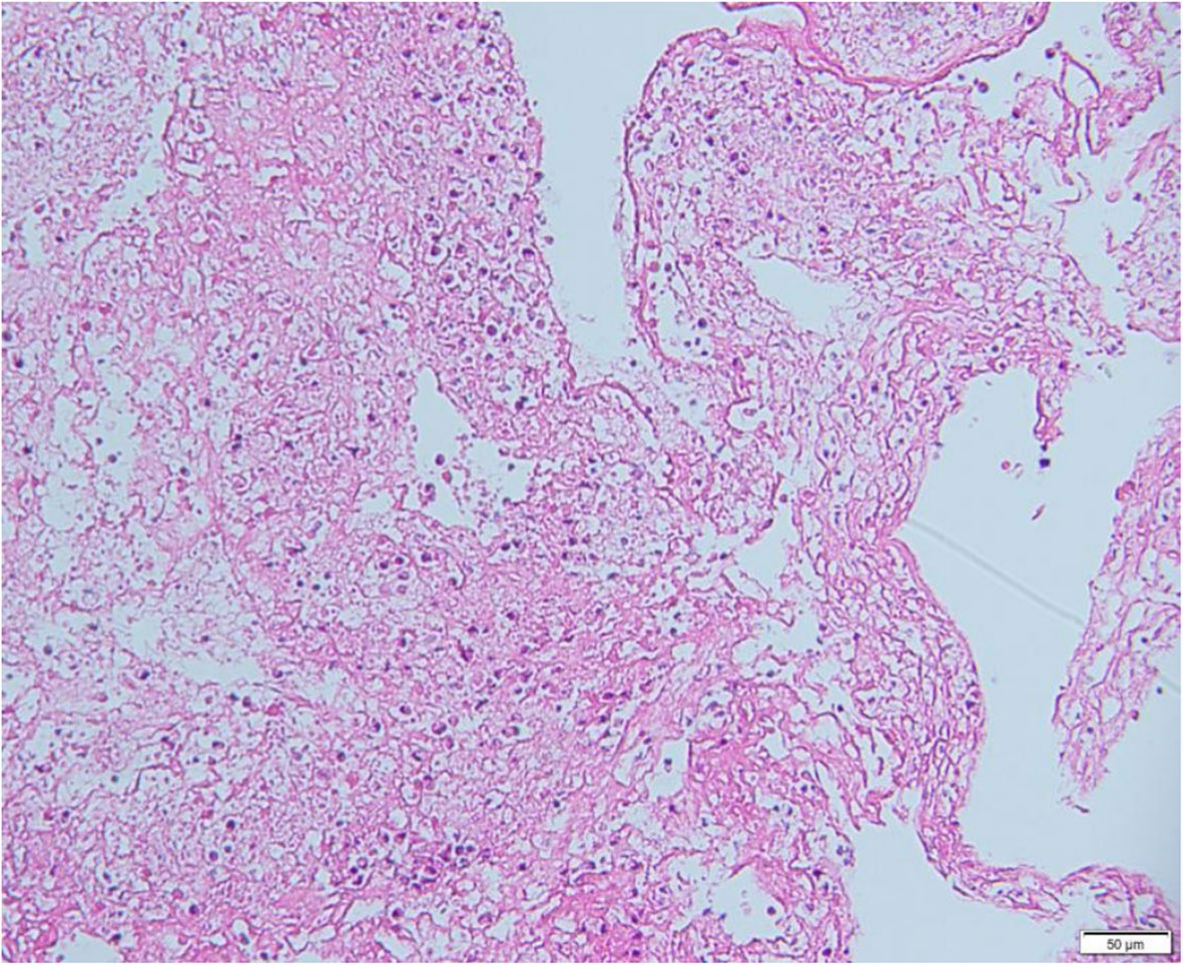
Pathological examination of mucus plugs or casts: Infiltration by a large number of neutrophils, and necrosis and exfoliation of the epithelial cells

Mucosal hyperaemia and oedema were observed in 88 (95.6%) children. Mucosal pallor was observed in 4 cases, with 1 case in the group with mucus plugs and 3 cases in the group without. Mucus plugs were found under fiberoptic bronchoscope, mainly in bilateral lower lobes (16 cases in the left lower lobe and 17 cases in the right lower lobe), which was consistent with the findings of pulmonary imaging. In 17 cases, the removed mucus plugs appeared like plastic casts, resembling a plastic-like material.(Mucous plugs and plastic casts are shown in Figs. 2 and 3. Pathological images of mucus plugs were shown in Fig. 4).

### Treatment

All children were nebulized for sputum. Anti-infective treatment was given until the pathogenetic results were returned. After the diagnosis was confirmed, antibiotics were stopped. Some children were considered to have a strong immune response due to the long duration of fever, and were treated with gammaglobulin to regulate immunity and low-dose methylprednisolone for anti-inflammatory treatment. All children were given fiberoptic bronchoscopy and alveolar lavage to clear respiratory secretions. Among them, 9 cases (42.8%) in the mucus plug group underwent two or more bronchoscopic alveolar lavage; 6 cases (15.7%) in the non-mucus plug group underwent two or more bronchoscopic alveolar lavage, and all of them were discharged after improvement.

### Multivariate logistic regression analysis and ROC curve results

**Fig. 5:**
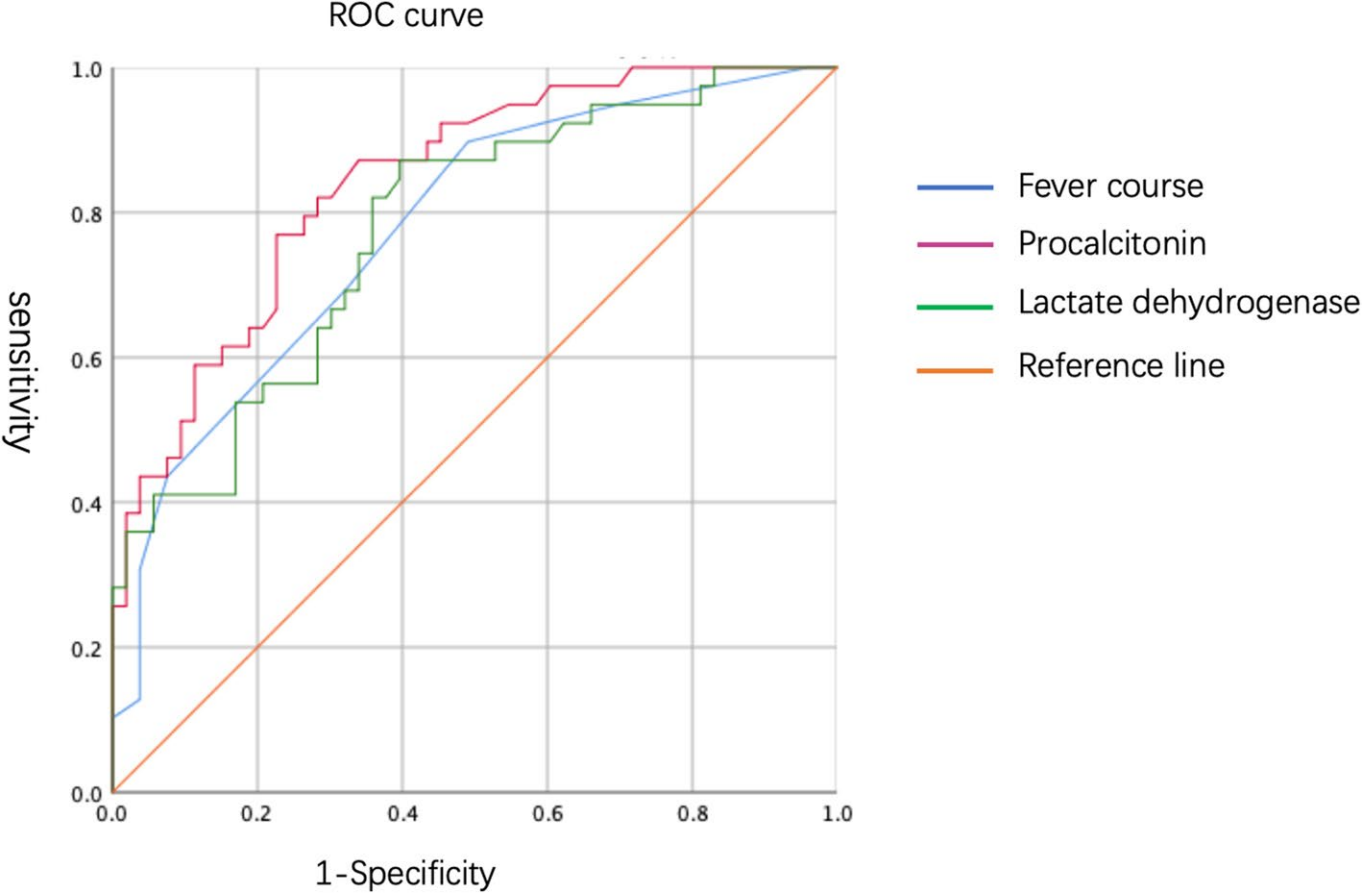
ROC curves of risk factors for the airway mucus plugs in children with ADV pneumonia

Multivariate logistic regression analysis was conducted for the statistically significant variables, i.e. duration of fever, PCT and LDH levels, pleural effusion and reduced breath sounds. The results showed that duration of fever, and the PCT and LDH levels were independent predictors of the development of mucus plugs, as shown in Table 3. Then, the ROC curves for these variables were plotted (Fig. 5). The cut-off values are as follows: for the duration of fever, value was ≥ 6.5 d, for PCT, level was ≥ 0.705 ng/ml, and for LDH, it was ≥ 478.5U/L (see Table 4).

**Table 3:** Logistic regression analysis of factors associated with the development of airway mucus plugs in children with ADV pneumonia

Indicator	B	SE	Wald *X*^2^ value	*P*-value	OR	95% CI
Fever duration	0.376	0.165	5.201	0.023	1.456	1.054–2.012
PCT	0.777	0.312	6.201	0.013	2.175	1.180–4.010
LDH	0.002	0.001	5.377	0.020	1.002	1.000–1.004
Diminished respiration	0.590	0.682	0.748	0.387	1.804	0.474–6.866
Pleural effusion	-0.234	0.686	0.117	0.733	0.791	0.206–3.035
Constant	-5.816	1.439	16.329	0.000	0.003	–-

*B* regression coefficient, *SE* standard error, *OR* odds ratio, *CI* confidence interval, *PCT* procalcitonin, *LDH* lactate dehydrogenase

**Table 4:** ROC curve thresholds for the predictive indicators of the development of airway mucus plugs in children with ADV pneumonia

Indicator	Threshold value	Sensitivity	Specificity	Area under the curve	*P*-value	95% CI
Fever duration	6.5d	89.7%	50.9%	0.778	< 0.01	0.684–0.873
PCT	0.705 ng/ml	76.9%	77.4%	0.843	< 0.01	0.764–0.921
LDH	478.5U/L	87.2%	60.4%	0.777	< 0.01	0.683–0.872

*CI* confidence interval, *PCT* procalcitonin, *LDH* lactate dehydrogenase

## Discussion

Bronchial mucus thrombi, first reported by Shaw in 1951, are one of the most important endogenous foreign bodies of the respiratory tract. They are usually formed due to inflammation, necrosis, haemorrhage and abnormal mucus secretion by the bronchial mucosa, resulting in mucus accumulation and plugging of the bronchi [4]. Current studies have found that a tracheal mucus plug, in children, is often associated with severe and refractory bronchopneumonia, uncontrolled bronchial asthma, and other respiratory diseases [5, 6]. However, due to an increased incidence of ADV pneumonia, the number of children presenting with airway mucus plug formation has also increased. This is thought to be related to the pathogenicity of the ADV. ADV causes the release of the inflammatory mediators that, in turn, cause increased mucus secretion, mucosal oedema, hyperaemia, necrosis and shedding, leading to the obstruction of the lumens by the necrotic material [3]. The present study compared the groups presenting with and without the mucus plugs, and found that among the 14 possible factors, only the fever duration, PCT and LDH levels were the three independent risk factors for the formation of airway mucus plugs in children with ADV pneumonia.

The mean duration of fever was significantly longer in the children with mucus plugs than those without. Prolonged fever indicates that the inflammation is not effectively controlled. Prolonged inflammatory stimulation can cause the release of a large number of inflammatory mediators and the activation of several cytokines (TNF-α, IL-1, IL-6, IL-8, IL-12, INF, etc.) [9], resulting in bronchial and fine bronchial mucosa congestion and oedema, necrotic shedding, and obstruction of the lumen by necrotic material, as well as increased mucus secretion and obstruction of the lumen [3], thus promoting the formation of mucus thrombi.

Among the 92 children enrolled in the study there were 54 cases where PCT was greater than 0.5 ng/ml, including 23 cases (43.3%) in the non-mucosal embolus group and 31 cases (79.4%) in the mucosal embolus group, and the mean level of PCT in the mucosal embolus group was significantly higher than that in the non-mucosal embolus group. This finding is inconsistent with the traditional belief that procalcitonin levels can be used as an indicator to identify bacterial and viral infections [10]. Relevant literature has pointed out that the procalcitonin level has no clinical value in the identification of bacterial and viral infections [11]. Some scholars believe that the serum PCT level in children with ADV pneumonia is significantly higher than in those with other types of respiratory viral pneumonia; furthermore, the PCT level in children with severe ADV pneumonia (especially type 3 and type 7) is higher than that in children with a mild ADV infection. The possible mechanism is thought to be related to the strong immune stimulation following the adenovirus infection, which leads to a significant increase in serum procalcitonin through the activation of the cytokines [12, 13]. According to Kawasaki [14] and Song [15], the immune response and cytokine activity is stronger after the adenovirus infection when compared to infections caused by the other respiratory viruses. Therefore, there might be some basis for the PCT level acting as a risk factor for the airway mucus plug formation in the children with ADV pneumonia.

Serum LDH is a glycolytic enzyme that is abundantly found in the myocardium, the skeletal muscles and the kidneys, as well as the liver, the pancreas, the lungs, etc. It can induce lactic acid production from pyruvic acid. It is released into the blood when either the myocardium or the other organs are injured, and its concentration in the blood increases rapidly. Because it is widely found in various tissues and organs of the human body, it has a low specificity (the specificity in this study was 60.4%). Still, in many diseases, LDH levels are used as a reliable indicator of the severity of illness and its prognosis [16, 17]. Serum LDH levels are also elevated in the presence of pulmonary inflammation and hypoxia. In this study, the LDH levels of the group with the mucus plugs were also significantly higher than those for the group without it. The inflammatory response of the lung and the degree of hypoxia were more severe in the group with the mucus plugs than in the group without it, and the cell membrane permeability was increased during hypoxia.

In addition, in the present univariate analysis, it was found that the children in the group with mucus plugs had a significantly higher proportion of complicated pleural effusion compared with the children in the other group. The fluid volume was low, and in most cases, the parapneumonic effusion was unilateral, which is consistent with the findings of the study done by Shen et al. [18]. However, the logistic regression equation could not be included, because of the small sample selection and the interaction among the single factors; therefore, large sample data are needed for further investigation. In addition, it was found that more children in the group with mucus plugs underwent two or more rounds of bronchoscopic alveolar lavage than those in the group without it. This was associated with heavy lung injury, difficulty in treatment, and difficulty in removing the secretions in the children with mucus plugs. Therefore, in children suspected of having mucus plugs or bronchial casts, early fiberoptic bronchoscopic alveolar lavage as an adjuvant therapy can help in the improvement of local ventilation and the removal of secretions. It can also accelerate the repair of the diseased mucosa, improve the prognosis, and reduce the occurrence of bronchiolitis obliterans [19–21].

In summary, for children with ADV pneumonia and a fever course of ≥ 6.5 days, PCT ≥ 0.705 ng/ml, and LDH ≥ 478.5 U/L, it is necessary to be vigilant in case of the formation of airway mucus plugs. However, since this study is a single-center study with a small sample size and a retrospective study, it will have an impact on the statistical results and needs to be explored in depth by multi-center or further expansion of the number of cases.The mechanism of development of ADV bronchial mucus plugs is still unclear. It may be related to the inflammatory and immune responses induced by the ADV infection, but this needs to be further investigated. As airway mucus plug lacks clinical specificity on imaging, it may lead to adverse sequelae if not removed promptly. Therefore, physicians should stay alert regarding the possible development of this condition. For children with ADV pneumonia, who are suspected of developing airway mucus plugs, fibreoptic bronchoscopy and alveolar lavage should be actively performed, in addition to the initiation of the symptomatic and antibiotic/antiviral therapy. Prompt bronchoscopy as an adjuvant therapy to remove respiratory secretions helps in unblocking the airway, reducing long-term complications, and improving the children's prognosis.

## Data Availability

The data used to support the findings of this study are available from the corresponding author upon request.
